# Viability and integrity of *Pinus densiflora* seeds stored for 20 years at three different temperatures

**DOI:** 10.1093/conphys/coae046

**Published:** 2024-07-09

**Authors:** Da-Eun Gu, Sim-Hee Han, Kyu-Suk Kang

**Affiliations:** Forest Bioresources Department, National Institute of Forest Science, Onjeong-ro 39, Gwonseon-gu, Suwon 16631, Republic of Korea; Department of Agriculture, Forestry, and Bioresources, Seoul National University, Kwanak-ro 1, Kwanak-gu, Seoul 08826, Republic of Korea; Forest Bioresources Department, National Institute of Forest Science, Onjeong-ro 39, Gwonseon-gu, Suwon 16631, Republic of Korea; Department of Agriculture, Forestry, and Bioresources, Seoul National University, Kwanak-ro 1, Kwanak-gu, Seoul 08826, Republic of Korea

**Keywords:** Carbohydrate metabolism, electrolyte leakage, Pinus seed, seed aging, seed storage, storage temperature

## Abstract

Storage temperature is one of the most important factors determining seed longevity in the genebank. This study aimed to investigate the effect of storage temperature on the seed viability and physiological integrity after a 20-year storage period of *Pinus densiflora*, a tree species of ecological and economic significance in South Korea. To this end, seeds were collected and stored dry for 20 years at −18°C, 4°C and 25°C. Germination tests were conducted to assess seed viability and vigour, electrolyte leakage analysis was performed to assess cell membrane integrity, and carbohydrate analysis was conducted to assess metabolic integrity during germination. The results revealed that over 20 years, seeds stored at −18°C maintained a high germination percentage (GP; 89%), comparable to initial GP (91%), whilst those stored at 4°C exhibited a decline in GP (44%) along with a decrease in vigour. Seeds stored at 25°C lost their viability entirely. Electrical conductivity of the leachate and leakage of inorganic compounds and soluble sugars were higher with elevated storage temperature, indicating increased imbibition damage. Additionally, changes in carbohydrate content during germination revealed that the loss of viability according to storage temperature is associated with reduced storage reserve utilization and altered carbohydrate metabolism during germination. These results enhance our understanding of the effect of seed storage temperature on longevity and physiological changes of aging in the genebank, serving as a reference for establishing conservation strategies for *Pinus densiflora*.

## Teaser

Storage temperature is an important factor determining the storability of seeds. We investigated the effect of storage temperature on the viability and physiological integrity of Pinus densiflora seeds after a 20-year storage. Our findings will provide valuable insights for seed conservation strategies.

## Introduction

Seeds, inherently cryptobiotic in a dry state, inevitably lose their viability undergoing an imperceptible transition from being viable to non-viable (Fleming *et al.*, 2019). To decelerate seed aging during seed storage, it is essential to minimize respiration and other metabolic activities (Colville and Pritchard, 2019). Consequently, reducing seed moisture content as much as possible and maintaining low storage temperatures are pivotal for seed preservation (Roberts and Ellis, 1989; Ellis, 1991; Bonner and Karrfalt, 2008). The tolerance to desiccation varies within and amongst species (Ballesteros *et al.*, 2020). Orthodox seeds, for example, acquire desiccation tolerance during development, enabling them to withstand the loss of most bound water and survive extended periods in a dry state (Pritchard, 2004; El-Maarouf-Bouteau, 2022). Although desiccation tolerance significantly extends seed shelf life, seeds ultimately lose viability and fail to produce normal seedlings due to aging. Studies have extensively examined physiological changes in seed aging, including increases in reactive oxygen species and oxidative stress, membranes becoming leaky, loss of enzymatic activity, degradation of storage proteins, and deoxyribonucleic acid and ribonucleic acid (Walters, 1998; Bertram and Hass, 2008; Rajjou *et al.*, 2008; Pukacka *et al.*, 2009; Zhang *et al.*, 2015; Fleming *et al.*, 2019). These aging mechanisms vary substantially amongst taxa, species and even amongst populations (Walters *et al.*, 2005; Gianella *et al.*, 2022). Because understanding these mechanisms is critical for predicting seed longevity and ensuring efficient genebank management, continuous research on the seed aging physiology of species targeted for conservation is necessary.

Seed aging rate is profoundly influenced by moisture content and storage temperature (Ellis and Roberts, 1980; Walters, 1998). Even dried seeds differ in the duration of maintaining viability depending on their inherent lifespan and storage temperature (Walters *et al.*, 2005). However, few studies directly compared seed viability at different storage temperatures in genebanks (van Treuren *et al.*, 2018; Chau *et al.*, 2019; Colville and Pritchard, 2019), and especially studies analyzing the physiological aspects of aging at various storage temperatures are scarce. Furthermore, most research on seed aging has used accelerated aging tests or controlled deterioration tests (Yaklich, 1985; Bernal-Lugo and Leopold, 1998; Han *et al.*, 2006; Lehner *et al.*, 2008; Kim and Han, 2010; Kim *et al.*, 2011; Gerna *et al.*, 2022), which use high relative humidity and temperature, and thus there may be discrepancies between the actual aging mechanisms and predicted longevity (Schwember and Bradford, 2010; Roach *et al.*, 2018; Colville and Pritchard, 2019; Gianella *et al.*, 2022). Therefore, to understand the physiology of seed aging in actual genebanks, it is appropriate to study seeds that have slowly lost viability after being stored for a long time in a low temperature environment.

*Pinus densiflora* Siebold & Zucc. is an evergreen coniferous tree distributed in the mountains below 1300 metres in the Korean Peninsula, China and Japan. *P. densiflora* holds substantial value for both timber and pine mushroom production. Beyond its economic significance, this species has significantly influenced the traditional lifestyle, culture and art in the Korean Peninsula. Due to these factors, the silvicultural demand remains consistently high, leading to its inclusion in 15.0% of South Korea’s total reforestation area over the last five years (Korea Forest Service, 2023). Despite being one of the most important economic and cultural tree species in Korea, its distribution area is decreasing due to increasing frequency of diseases such as pine wilt, wildfire and ecological succession (Bae *et al.*, 2012). Therefore, effective genebank management for *ex situ* conservation and restoration of *P. densiflora* is required, necessitating continuous data collection and research on the aging physiology of long-term stored seeds.

Accordingly, this study aimed to compare the viability and physiological integrity of *P. densiflora* seeds aged by long-term genebank storage under different storage temperatures, focusing on electrolyte leakage during imbibition and carbohydrate metabolism during germination. To this end, *P. densiflora* seeds stored for 20 years at −18°C, 4°C and 25°C underwent comprehensive experiments to answer the following research questions: (i) how do germination characteristics vary amongst seeds stored at different temperatures, and how long can seeds maintain their viability and vigour at each temperature? (ii) How does electrolyte leakage during imbibition, which implies changes in cell membrane integrity, vary in seeds stored under different temperatures? (3) How are glucose, sucrose and starch utilized during various germination stages in seeds stored at different temperatures, and what can be inferred about their metabolic activities?

## Materials and Methods

### Seed collection and storage

The *P. densiflora* cones were collected in October 2003 from an open-pollinated clonal seed orchard in Anmyeondo, Republic of Korea. Following collection, the cones were randomly mixed in bulk and dried in the shade of a greenhouse for a week, and the seeds were subsequently extracted and vacuum-packed in aluminium foil pouches for storage. The seeds were stored for 20 years at the seed storage facility of the National Institute of Forest Science in the Republic of Korea, under three different standard seed storage temperature (FAO, 2014): −18°C (standard for long-term storage), 4°C (standard for medium-term storage) and 25°C (standard for short-term storage). At the time of storage, the seed moisture content was 4.8 ± 0.6%, and the initial germination percentage (GP) was 91 ± 1.5%.

### Germination test and calculation of germination characteristics

To investigate the viability and vigour of seeds stored at three temperatures for 20 years, germination test was conducted, and indices representing germination characteristics were calculated. For the germination tests, a 90 mm Petri dish with two filter papers (Whatman No. 2) moistened with distilled water was used as the germination medium, and 25 seeds soaked in water for 24 hours were placed on it in four replicates (ISTA, 2020). The seeds were incubated in a growth chamber set to 24°C, with 16 hours of light and 8 hours of darkness, and germination was monitored daily until no further germination occurred. Seeds with radicles grown more than 2 mm were considered germinated. Seeds were not removed until they grew into seedlings, and the number of seeds that produced normal seedlings was counted (ISTA, 2020).

Using the germination data, the GP, *t*_50_ (the number of days required to achieve 50% of the final GP; Farooq *et al.*, 2005), mean germination time (MGT) (Ranal and de Santana, 2006), germination speed (GS) (McNair *et al.*, 2012), and the coefficient of uniformity of germination (CUG) (Ranal and de Santana, 2006) were calculated as follows:

–
  $$ \mathrm{GP}=\frac{N}{S}\times 100\ \left(\%\right) $$
–
  $$ {T}_{50}=\frac{\left(\frac{N}{2}-{N}_i\right)\left({T}_j-{T}_i\right)}{\left({N}_j-{N}_i\right)}\ (days) $$
–
  $$ \mathrm{MGT}=\frac{\sum_{i=1}^k{N}_i{T}_i}{N}\ (days) $$
–
  $$ \mathrm{GS}=\sum_{i=1}^k\frac{N_i}{T_i}\ \left( seeds/ day\right) $$
–
  $$ \mathrm{CUG}=\frac{\sum_{i=1}^k{N}_i}{\sum_{i=1}^k{\left( MGT-{T}_i\right)}^2{N}_i} $$

(S: total number of tested seeds; N: final number of germinated seeds; N_i_: cumulative number of seeds that germinated just before reaching 50% of final germination; N_j_: cumulative number of seeds that germinated just after 50% of final germination; T_i_, T_j_: number of days taken to reach N_i_ and N_j_ points, respectively)

### Analysis of electrolyte leakage characteristics

To evaluate the integrity of cell membranes, the electrolyte leakage characteristics of seeds stored at three temperatures for 20 years were analyzed. This involved measuring the electrical conductivity of the seed leachate and analyzing inorganic compounds and soluble sugars in the leachate. To analyze electrical conductivity and inorganic compounds, 25 seeds in four replicates were washed more than 10 times to remove surface contaminants and immersed in 25 ml of ultrapure water per replicate. They were agitated on a shaker at 110 rpm in dark conditions for 24 hours. Afterward, the seeds were separated from the leachate and used for the germination tests described earlier. The electrical conductivity of the leachate was measured using an Orion Star A215 pH/Conductivity Benchtop Multiparameter Meter (Thermo Fisher Scientific Inc., Waltham, MA, USA). Inorganic compounds analysis was conducted using a PlasmaQuant PQ 9000 Elite High-resolution Array ICP-OES (Analytik Jena AG, Jena, Germany) to determine Na, Mg, K, Ca, Fe and Cu in the leachate.

To examine soluble sugar leakage during seed imbibition, glucose and sucrose in the leachate were analyzed. Considering the expected low concentrations, 25 seeds were soaked in 2 ml of ultrapure water in triplicate, rotated on a shaker at 110 rpm for 48 hours, and filtered through Whatman No. 2 filter paper. The glucose and sucrose were analyzed using the glucose oxidase method (Armstrong *et al.*, 1993).

### Carbohydrate analysis

To assess the integrity of carbohydrate metabolisms during germination of seeds stored at three temperatures for 20 years, the contents of glucose, sucrose and starch at different germination stages were analyzed. The germination process was divided into distinct stages: Stage I, dry state; Stage II, imbibition (24 hours of imbibition); Stage III, pre-germination (4 days of incubation); and Stage IV, after germination (radicle emergence). Seeds stored at 25°C, which did not germinate, were analyzed only for Stages I, II and III. For analysis, more than 400 seeds were placed in the same germination medium as used in the germination test and sampled at each germination stage. The seed coats were removed from the sampled seeds, which were then freeze-dried for 72 hours. After freeze-drying, the seeds were ground with a mortar and pestle in liquid nitrogen. The ground samples were prepared in amounts of at least 0.5 g for each treatment, and then 0.1 g from each batch was used for four replicate measurements. Each replicate was homogenized with 1.5 ml of extraction solvent (methanol: chloroform: H2O in the ratio 12: 5: 3) and centrifuged at 13 000 x g at 24°C for 4 minutes. The supernatant was used for glucose and sucrose analysis, and the precipitate was used for starch analysis. The glucose, sucrose and starch contents were determined using the glucose oxidase method (Armstrong *et al.*, 1993).

### Statistical analysis

Germination characteristic indices were calculated using a user-defined function of R software version 4.2.2 (R Core Team, 2023). One-way analysis of variance (ANOVA) and Duncan’s multiple range test were employed to identify differences amongst storage temperatures in germination characteristics, electrical conductivity, inorganic compound concentrations and carbohydrate contents at different germination stages, respectively. One-way ANOVA was performed using the ‘anova’ function in R software version 4.2.2 (R Core Team, 2023), and Duncan’s multiple range test was conducted with the ‘duncan.test’ function in the ‘agricolae’ package (Mendiburu, 2023).

## Results

### Effects of seed storage temperature on germination characteristics

The germination characteristics of *P. densiflora* seeds stored for 20 years at three different temperatures are presented in Table 1. Excluding the CUG, significant differences were observed in GP, *t*_50_, MGT and GS amongst the seeds stored at the three different temperatures (*P* < 0.05, Table 1). For seeds stored at −18°C, the average GP was highest (89%), similar to the initial GP before storage (91%), indicating minimal loss in viability. Additionally, these seeds exhibited the most rapid germination, as evidenced by the shortest *t*_50_ (6.0 days) and the highest GS (3.6 seeds per day), suggesting superior seed vigour. However, seeds stored at 4°C showed an average GP of 44%, and delayed germination with *t*_50_ and MGT averaging 8.5 and 9.9 days, respectively, indicating a decrease in both seed viability and vigour. Seeds stored at 25°C did not germinate at all, showing a complete loss of viability.

**Table 1:** Germination characteristics according to seed storage temperature

Storage temperature (°C)	GP (%)	Percentage of normal seedlings (%)	*T*_50_ (day)	MGT (day)	GS (seeds/day)	CUG
-18	89 ± 4.4^a^	80 ± 4.9 ^a^	6 ± 0.4 ^b^	6.8 ± 0.5 ^b^	3.6 ± 0.1 ^a^	0.29 ± 0.12
4	44 ± 5.4^b^	22 ± 6.0 ^b^	8.5 ± 0.3 ^a^	9.9 ± 0.3^a^	1.3 ± 0.2^b^	0.08 ± 0.04
25	0 ± 0^c^	0 ± 0^c^	—	—	—	—
F-value	121.24	85.40	26.42	27.70	150.91	2.91
*P*-value	<0.0001^***^	<.0001^***^	0.0021^**^	0.0019^**^	<0.0001^***^	0.1388^NS^

Each field value represents mean ± standard error (*n* = 4).

NS, ** and *** are not significant; significant at *P* < 0.01, and *P* < 0.001, respectively.

Different letters in a column represent significant differences amongst groups according to Duncan’s multiple range tests at α = 0.05, with letters assigned in order of mean values.

### Effects of seed storage temperature on electrolyte leakage characteristics

#### Electrical conductivity of seed leachate

**Figure 1:**
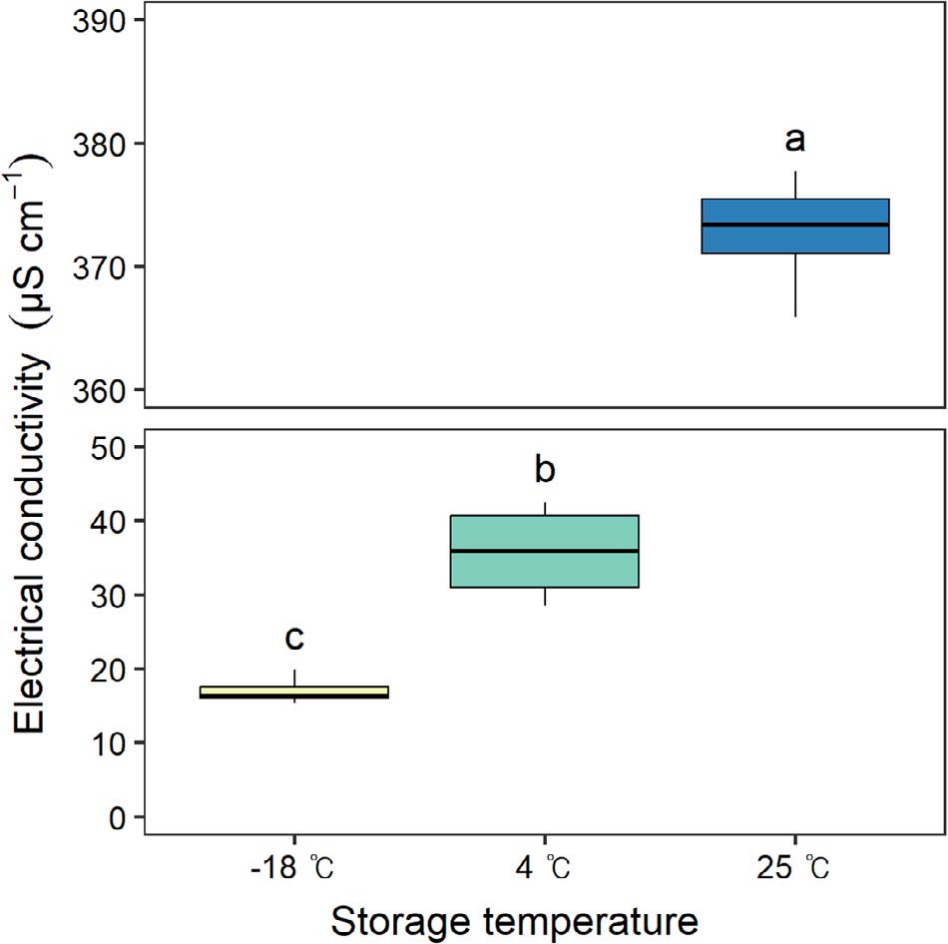
Electrical conductivity of seed leachate according to seed storage temperature. Different letters above the box-and-whisker plots represent significant differences amongst storage temperatures according to Duncan’s multiple range test at α = 0.05, with letters assigned in order of mean values.

There were significant differences in electrical conductivity amongst the seeds stored for 20 years at three different storage temperatures (*P* < 0.05, Fig. 1). The electrical conductivity was highest for seeds stored at 25°C (372.9 ± 1.0 μS cm^−1^), followed by 4°C (35.9 ± 1.6 μS cm^−1^) and − 18°C (17.0 ± 0.5 μS cm^−1^), indicating a strong relationship between the electrical conductivity of seed leachate and seed viability.

#### Inorganic compounds in seed leachate

**Figure 2:**
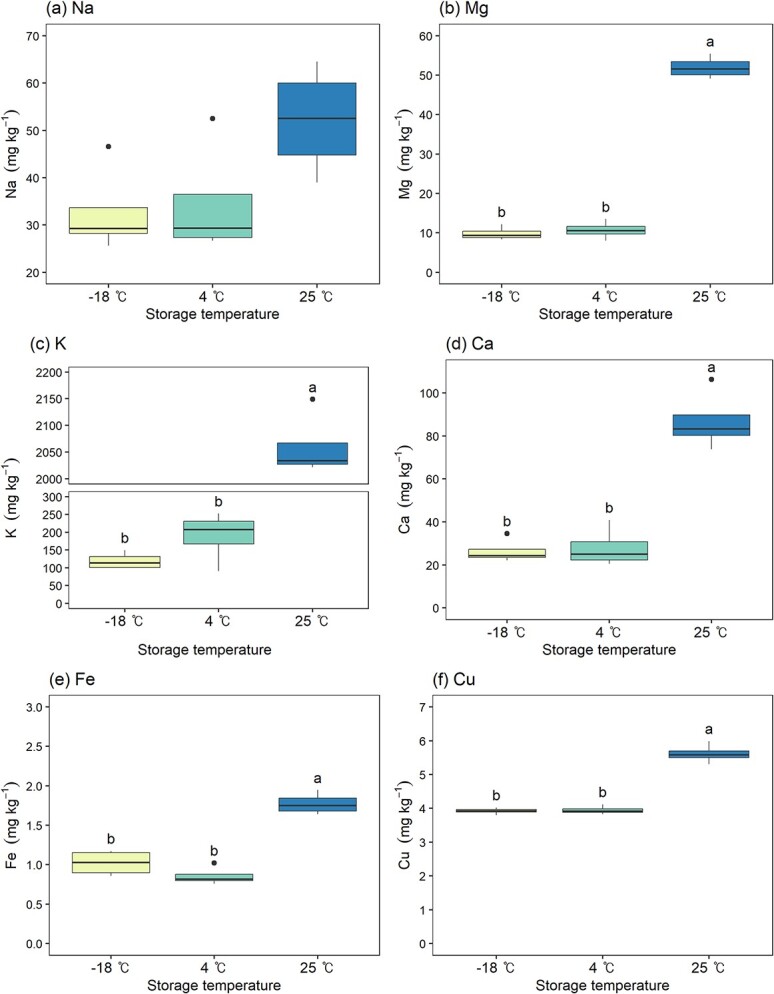
Concentration of inorganic compounds in seed leachate according to seed storage temperature. Different letters above the box-and-whisker plots represent significant differences amongst storage temperatures according to Duncan’s multiple range test at α = 0.05, with letters assigned in order of mean values. Box-and-whisker plots without letters indicate that there was no significant difference amongst storage temperatures according to ANOVA at α = 0.05.

The concentration of inorganic compounds in the seed leachate is shown in Fig. 2. There were significant differences in the concentrations of Mg, K, Ca, Fe and Cu amongst the seeds stored for 20 years at three different storage temperatures (*P* < 0.05). All detected inorganic compound concentrations were highest in seeds stored at 25°C. For Mg, K, Ca, Fe and Cu, there were no significant differences between the −18°C and 4°C, but the values were significantly higher in seeds stored at 25°C. The concentration of K, the most detected element, tended to increase as the seed viability and vigour decreased. Although not statistically significant, the concentration of Na was highest in seeds stored at 25°C.

#### Soluble sugars in seed leachate

**Figure 3:**
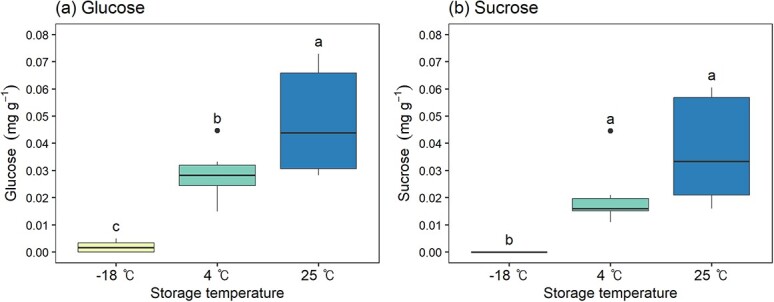
Concentration of soluble sugars in seed leachate according to seed storage temperature. Different letters above the box-and-whisker plots represent significant differences amongst storage temperatures according to Duncan’s multiple range test at α = 0.05, with letters assigned in order of mean values.

There were significant differences in the leaked glucose and sucrose content amongst the seeds stored for 20 years at three different temperatures (*P* < 0.05, Fig. 3). Seeds stored at −18°C showed minimal glucose leakage (0.002 ± 0.001mg g^−1^) with no detectable sucrose. Seeds stored at 4°C and 25°C exhibited higher levels of leaked glucose and sucrose compared to the seeds stored at −18°C. At 4°C, glucose measured 0.029 ± 0.004 mg g^−1^, and sucrose was detected at 0.021 ± 0.005 mg g^−1^. At 25°C, both glucose (0.048 ± 0.008 mg g^−1^) and sucrose (0.037 ± 0.008 mg g^−1^) levels were the highest amongst the storage temperatures, indicating an upward trend in leaked soluble sugar content with increasing storage temperature.

### Effects of seed storage temperature on changes in carbohydrate content during germination

**Figure 4:**
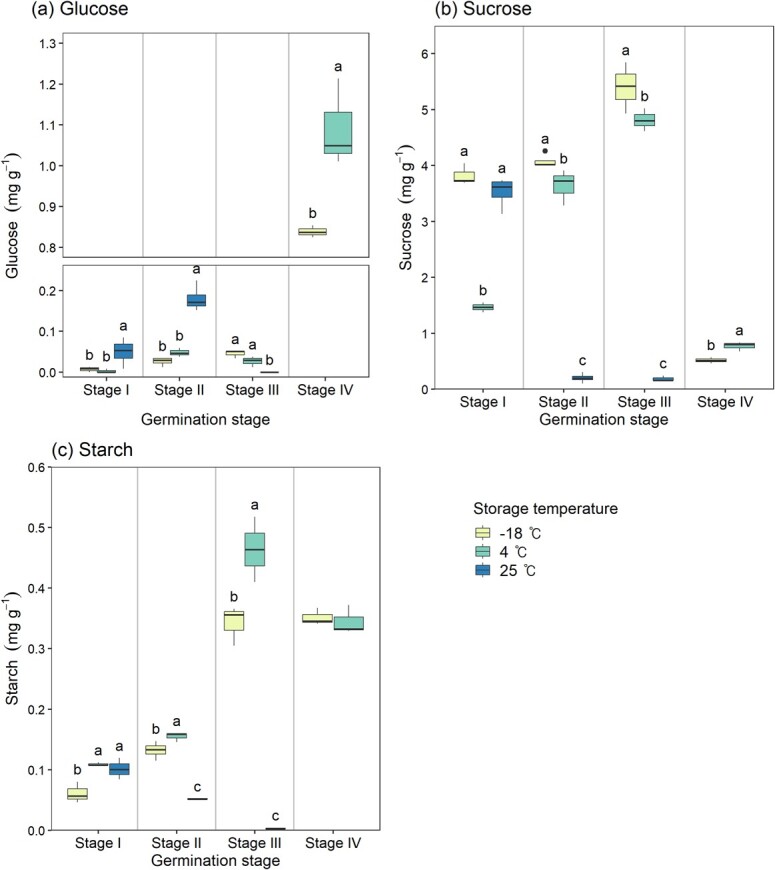
Carbohydrate contents at different germination stages according to seed storage temperature. Different letters above the box-and-whisker plots represent significant differences amongst storage temperatures according to Duncan’s multiple range test at α = 0.05, with letters assigned in order of mean values. Box-and-whisker plots without letters indicate that there was no significant difference amongst storage temperatures according to ANOVA at α = 0.05. Stage I: dry state; Stage II: imbibition; Stage III: pre-germination; Stage IV: after germination.

Figure 4 displays the carbohydrate content at different germination stages of the seeds stored for 20 years at three different temperatures. In the dry state, sucrose was the most abundant carbohydrate amongst the measured carbohydrates, and it remained the highest at both the imbibition and pre-germination stages, regardless of storage temperatures. However, after germination, there was a significant decrease in sucrose content, accompanied by an increase in glucose.

The glucose content exhibited significant differences amongst storage temperatures across all germination stages (*P* < 0.05, Fig. 4a). For the seeds stored at −18°C and 4°C, the glucose content started increasing from the imbibition stage and was highest after germination. However, for the seeds stored at 25°C, the highest value (0.180 mg g^−1^) was at the imbibition stage, which was higher than other storage temperatures, but dropped to 0 just before germination.

The sucrose content also showed clear differences amongst storage temperatures at all germination stages (*P* < 0.05, Fig. 4b). It was highest in the seeds stored at −18°C in all stages. For the seeds stored at −18°C and 4°C, the sucrose content increased from the imbibition stage, peaked just before germination, and then decreased sharply after germination. However, for the seeds stored at 25°C, it showed the highest value at the dry state (3.526 mg g^−1^) and then decreased sharply as the seeds absorbed water (0.201 mg g^−1^) and germination progressed (0.187 mg g^−1^).

The starch content showed differences in the dry state, imbibition, and pre-germination stages depending on storage temperatures (*P* < 0.05, Fig. 4c). In the dry state, the starch content in the seeds stored at 4°C (0.109 mg g^−1^) and 25°C (0.101 mg g^−1^) showed no difference, and it was lowest in the seeds stored at −18°C (0.075 mg g^−1^). The starch content in the seeds stored at −18°C and 4°C, similar to glucose and sucrose, increased from the imbibition stage, peaked pre-germination stage and then decreased after germination. However, for the seeds stored at 25°C, although the starch content in the dry state (0.101 mg g^−1^) was similar to other seeds, it decreased sharply (0.052 mg g^−1^) from the imbibition stage, and dropped close to 0 (0.003 mg g^−1^) just before germination. The starch content after germination showed no difference between the seeds stored at −18°C and 4°C.

## Discussion

### Viability and vigour according to seed storage temperature

The most significant factors determining the ability of seeds to survive during storage are the storage temperature, seed moisture content, and inherent lifespan of the seeds. There are 151 *Pinus* taxa registered in the seed information database of the Society for Ecological Restoration, and storage behaviour information is provided for 65 of these species. According to this information, most *Pinus* seeds are classified as orthodox seeds, for which hermetic storage at a moisture content of 7%–9% is recommended. The storage longevity varies: it can be 10–20 years at −18°C, 5–20 years at 4°C and 1–7 years at room temperature (Society for Ecological Restoration *et al.*, 2023). For *P. densiflora* seeds, previous studies reported that they maintained viability for six years at −20°C (Society for Ecological Restoration *et al.*, 2023), and there was minimal viability loss in seeds stored at 4°C for 12 years, but another seed lot showed significant seed vigour reduction after being stored for 14 years under the same conditions (Gu *et al.*, 2022). Our study expands this research, directly comparing storability according to storage temperatures by collecting seeds from a single seedlot, drying them to a moisture content of 4.8% and storing them for 20 years at three standard temperatures for seed storage: −18°C, 4°C and 25°C.

Seed aging due to 20-year storage was evaluated by viability (as shown by GP) and vigour (as indicated by *t*_50_, MGT, GS and germination uniformity). Generally, vigour declines before viability in natural seed aging (Delouche and Caldwell, 1960). Because seed vigour is a concept including various aspects of seed performance such as GS and uniformity, stress resistance, storability and seedling emergence in the field, which are not evident from GP alone (Marcos-Filho, 2015; Finch-Savage and Bassel, 2016), it was considered to assess the degree of seed aging along with viability. The results indicated that seeds stored at −18°C maintained both their viability and vigour, whereas those stored at 4°C displayed reduced seed vigour, as evidenced by increased *t*_50_ and MGT, decreased GS, and a significant reduction in viability (44% GP). Complete viability loss was observed in seeds stored at 25°C. This suggests that storage temperature significantly affects the viability and vigour of *P. densiflora* seeds, and that dried seeds with a moisture content below 5% can retain almost all their viability and vigour at a freezing temperature even after two decades. In genebank management, the time taken for GP to drop to 50% of the initial value (P50) is sometimes used as an indicator of seed longevity (Walters *et al.*, 2005; Colville and Pritchard, 2019; Hay *et al.*, 2022). Although this study lacks time-course germination data for precise calculation, seeds stored at 4°C for 20 years still had a 44% GP, suggesting that the seed longevity defined by P50 is close to 20 years at refrigeration temperature. However, seeds, even when dried, lose viability completely at room temperature.

Collecting empirical, systematic data on seed viability is crucial for genebank management and predicting seed longevity; yet, this task is challenging due to the need for long-term storage and monitoring. This study demonstrates the impact of temperature on seed storage by comparing the storage longevity of seeds from the same seed lot stored at different temperatures, revealing the actual longevity of *P. densiflora* seeds. Given that seeds stored at −18°C retain significant viability, further monitoring is necessary. Moreover, similar data collection efforts should be extended to other species in genebanks.

### Membrane integrity according to seed storage temperature

Seeds storage at varying temperatures influenced not only viability but also electrolyte leakage during water absorption. Seeds stored at 25°C, which lost all viability, showed more than 10-fold higher electrical conductivity in the leachate. Previous studies on tree seeds, including genus *Pinus*, demonstrated similar correlations (Bonner and Vozzo, 1986; Ramos *et al.*, 2012; Takos *et al.*, 2012; Gu *et al.*, 2022). The increased conductivity of seed leachate is attributed to cell membranes integrity loss in aged seeds. Rapid water influx during imbibition can temporarily damage cell membrane, due to perturbation and pressure caused by the water potential differential. Although healthy seeds quickly recover from this damage (Lin *et al.*, 2019), non-viable seeds lose the ability to do so, resulting in leakage of cytoplasmic solutes such as soluble sugars, inorganic compounds, organic acids and amino acids (Simon and Harun, 1972; Mattews and Powell, 2006; Choi and Yang, 2015).

In examining the inorganic compounds leaked into the seed leachate, this study found that the majority of the inorganic compounds present were K, followed by Ca, Na, Mg, Cu and Fe. Previous research on the leaching characteristics of inorganic elements in aged seeds also reported that K, Na and Ca were the most abundant minerals detected in seed leachate. (Woodstock *et al.*, 1985; Dias *et al.*, 1997; Han *et al.*, 2006; Mattews and Powell, 2006; Kim and Han, 2010). Woodstock *et al.* (1985) reported that K and Ca leakage could be indicators of a seed’s physiological potential in his study on cotton seeds. Likewise, this study found the concentrations of Mg, K, Ca and Fe in the leachate varies according to seed viability. Although the specific roles of these inorganic elements in seed viability are not yet understood, their general functions in plant cells are well-known: K regulates osmotic pressure and acts as an activator for various enzymes, whilst Na, not typically essential for higher plants, can partially substitute for K in cases of deficiency. Ca stabilizes cell walls and is involved in cell elongation, and Mg, Fe and Cu serve as cofactors for stress-related proteins (Husted *et al.*, 2011; Eggert and Von Wirén, 2013; Maathuis, 2014). Thus, the leakage of these inorganic elements during imbibition might be related to the loss of viability in seeds.

Furthermore, the content of soluble sugars such as glucose and sucrose in seed leachate increased in ascending order of −18°C, 4°C and 25°C. This indicates that whilst viable seeds prevent leakage of soluble sugars when soaked in water, aged seeds stored at higher temperatures exhibit a greater tendency to leak these metabolically essential sugars.

In conclusion, this study confirmed that various inorganic compounds and soluble sugars are leaked during imbibition due to seed aging, leading to differences in the electrical conductivity of the leachate. These results imply that the leakage of essential elements, micronutrients and soluble sugars could be related to the loss of seed viability and vigour. Therefore, the electrical conductivity test and measurement of electrolyte can be used as a simple method to test seed aging indirectly. Future research elucidating the role of inorganic elements in seed germination and seedling growth could establish a more definite causal relationship.

### Carbohydrate metabolism according to storage temperature

Analysis of carbohydrate content at different germination stages of *P. densiflora* seeds stored at various temperatures revealed that sucrose was the predominant carbohydrate in all seeds during the dry state, imbibition and pre-germination stages. It is well-known that orthodox seeds, which can withstand dehydration and rehydration, accumulate sucrose and raffinose family oligosaccharides during development as part of a protective mechanism against cellular damage during imbibition (Chen and Burris, 1990; Leprince *et al.*, 1990; Steadman *et al.*, 1996). Given their desiccation tolerance and the demonstrated longevity of up to 20 years in this study, it is reasonable to infer that *P. densiflora* seeds inherently possess high sucrose content, as they are characterized as orthodox seeds. Sucrose forms intercellular glasses with other non-reducing sugars and specific proteins like late embryogenesis abundant proteins and small heat shock proteins (Buitink and Leprince, 2004; Bewley *et al.*, 2013). The transformation of cytoplasm into this glassy state is thought to be essential in maintaining structural integrity of the membrane and proteins during desiccation (Koster and Leopold, 1988; Chen and Burris, 1990; Steadman *et al.*, 1996; Bryant *et al.*, 2001; Buitink and Leprince, 2004; Ballesteros *et al.*, 2020). Thus, the accumulation of sucrose during development likely contributed to maintaining viability throughout the drying process and 20 years of storage.

However, seeds stored at 4°C showed about 3-fold lower sucrose content than the seeds stored at −18°C. Previous studies on artificially aged wheat (Lehner *et al.*, 2008), corn (Bernal-Lugo and Leopold, 1998) and soybean (Yaklich, 1985) seeds reported that hydrolytic enzymes like α-galactosidase and invertase act during artificial aging, leading to decreased raffinose and sucrose levels (Lehner *et al.*, 2008). It is speculated that at 4°C storage conditions, although enzymatic activities and metabolic processes like respiration are slow, they still consumed sucrose. This reduction in sucrose content, necessary for maintaining desiccation tolerance, might partially lead to the observed loss of viability. Conversely, seeds that were slowly aged at 25°C showed no differences in soluble sugars like glucose and sucrose before water absorption, likely due to negligible metabolic activity in these dead seeds, unlike the rapid processes occurring during artificial aging in high temperature and humidity.

During the germination process in seeds with remaining viability (excluding those stored at 25°C), there was a gradual increase in sucrose content. Like most gymnosperms, *P. densiflora* seeds are oily seeds that primarily store lipids in the form of triacylglycerols, and those lipid reserves are converted to sugars during germination and utilized for embryo growth (Ching, 1966; Stone and Gifford, 1999). Therefore, the increase in sucrose content observed in viable seeds suggests that during imbibition, the lipid reserves were broken down and accumulated as sucrose, which is then utilized for the growth of the embryo. Also, the amount of sucrose in the −18°C and 4°C stored seeds corresponded with their viability levels, indicating more active conversion of storage reserves to sucrose in more viable seeds. However, seeds stored at 25°C, which completely lost their viability, showed a significant reduction in sucrose content during imbibition. This is postulated to be due to leakage of soluble sugars (Fig. 3) resulting from the loss of cell membrane integrity as discussed in the previous section, and a possible loss of relevant enzyme activity may also be a contributing factor.

After germination, −18°C and 4°C stored seeds showed a sharp decrease in sucrose and increase in glucose. This pattern aligns with the previous understanding that sucrose moves towards the embryo and is metabolized into simpler sugars like glucose and fructose by invertase within 10–20 hours after germination starts (Kuo *et al.*, 1990; Pukacka *et al.*, 2009).

Starch content did not show significant differences at the dry state across different storage temperatures but began to increase during imbibition. For the −18°C and 4°C stored seeds, starch content peaked just before germination and then decreased. This pattern of starch accumulation was also observed in other conifer’s oily seeds (Ching, 1966; Murphy and Hammer, 1994; Stone and Gifford, 1999). Murphy and Hammer (1994) suggested that during the development of a seed into a seedling, sucrose is not only used for respiration and biosynthesis but is also converted into starch, based on his observation that the pattern of starch accumulation in different organs of a seedling slightly lags behind the patterns of sucrose content and sucrose synthase activity. This starch accumulation in the developing seedling could be either a temporary storage deposit of sucrose, creating a sucrose gradient to facilitate its movement towards the embryo, or being used directly for growth (Li and Ross, 1990; Murphy and Hammer, 1994). In seeds stored at 4°C that had partially lost their viability, the accumulation of starch during germination occurred similarly to the seeds stored at −18°C. However, starch accumulation seemed not to occur in seeds that completely lost viability due to aging, evidenced by the results showing that the starch content actually decreased after imbibition in seeds stored at 25°C.

In summary, seeds stored at various temperatures display distinct patterns of carbohydrate changes during germination, depending on their respective viability levels. Seeds stored at −18°C exhibit patterns of glucose, sucrose, and starch changes consistent with the existing knowledge, which implies they maintain metabolic integrity. For seeds stored at 4°C, metabolic activities such as respiration during storage appear to have consumed some sucrose, and the patterns of glucose, sucrose and starch during germination are similar, but with lower accumulated sucrose compared to those at −18°C. Seeds stored at 25°C show no significant differences in carbohydrate content in the dry state compared to others but exhibit a significant decrease in glucose and sucrose during imbibition. This decrease is attributed to leakage during imbibition and the water absorption process, as confirmed by seed leachate analysis, leading to a reduction in content, and no additional synthesis seems to have occurred. Additionally, metabolic activities such as lipid utilization, the breakdown of non-reducing sugars, and starch accumulation do not occur in seeds that have completely lost viability due to aging. These observations imply that seeds utilize their storage reserves and undergo metabolic activities to varying extents, influenced by the degree of aging they have experienced.

## Conclusion

Storage temperature is a crucial factor in determining the storability of seeds for *ex situ* conservation. However, studies that directly compare the actual longevity of a species under different storage temperatures or explore the physiological aspects of aging in genebank at various temperatures remain limited. Our study revealed that for orthodox seeds like *P. densiflora*, seed viability and physiological properties due to aging are significantly influenced by storage temperature during long-term storage in a dry state. When other conditions are equal, *P. densiflora* seeds can maintain half of their viability after 20 years of storage at the typical refrigeration temperature of 4°C, whilst they retain almost all their viability and vigour at the freezing temperature of −18°C. Therefore, for long-term storage of *P. densiflora* seeds, drying them to a moisture content close to 5% and storing them at freezing temperature minimizes the loss of viability for up to 20 years. Furthermore, the period during which seed viability falls below 50% of its initial level, known as P50, appears to be nearly 20 years at 4°C.

Significantly, the study highlights that the variation in seed viability across different storage temperatures is due to changes in physiological properties caused by aging. Electrolyte leakage analysis revealed that higher storage temperatures lead to a diminished ability to recover cellular membranes and cause imbibition damage due to aging. Furthermore, differences in the carbohydrate content pattern during germination indicated that seed storage reserve utilization and carbohydrate metabolic activities are also influenced by storage temperature. These physiological aspects of seed aging will help in comprehensively understanding the impact of storage temperature on seed longevity and in establishing effective conservation strategies for *P. densiflora*. Moreover, further research on the physiological processes of seed aging and the development of biological markers will be a major advancement in understanding and predicting seed longevity, significantly improving conservation strategies for the seeds.

## Data Availability

The data underlying this article will be shared on reasonable request to the corresponding author.
